# The effects of breastfeeding on childhood BMI: a propensity score matching approach

**DOI:** 10.1093/pubmed/fdw093

**Published:** 2017-09-09

**Authors:** Laura A Gibson, Mónica Hernández Alava, Michael P Kelly, Michael J Campbell

**Affiliations:** 1 Health Economics and Decision Science, School of Health and Related Research, University of Sheffield, Sheffield S1 4DA, UK; 2 Institute of Public Health, University of Cambridge, Cambridge CB2 0SR, UK; 3 Design, Trials and Statistics, School of Health and Related Research, University of Sheffield, Sheffield S1 4DA, UK

**Keywords:** children, obesity, public health

## Abstract

**Background:**

Many studies have found a statistical association between breastfeeding and childhood adiposity. This paper investigates whether breastfeeding has an effect on subsequent childhood body mass index (BMI) using propensity scores to account for confounding.

**Methods:**

We use data from the Millennium Cohort Study, a nationally representative UK cohort survey, which contains detailed information on infant feeding and childhood BMI. Propensity score matching is used to investigate the mean BMI in children breastfed exclusively and partially for different durations of time.

**Results:**

We find statistically significant influences of breastfeeding on childhood BMI, particularly in older children, when breastfeeding is prolonged and exclusive. At 7 years, children who were exclusively breastfed for 16 weeks had a BMI 0.28 kg/m^2^ (95% confidence interval 0.07 to 0.49) lower than those who were never breastfed, a 2% reduction from the mean BMI of 16.6 kg/m^2^.

**Conclusions:**

For this young cohort, even small effects of breastfeeding on BMI could be important. In order to reduce BMI, breastfeeding should be encouraged as part of wider lifestyle intervention. This evidence could help to inform public health bodies when creating public health guidelines and recommendations.

## Introduction

Childhood obesity has increased in recent years and obese children may become obese adults^1^ and suffer from associated co-morbidities.^2,3^ Early-life factors could play a role in determining levels of childhood body mass index (BMI) and therefore future obesity levels in adults. If this is so, it has important policy implications; early-life interventions could help reduce later-life co-morbidities.

The effects of breastfeeding on childhood obesity have been debated in an extensive but inconclusive literature.^4–12^ Breastfeeding is known to have numerous benefits to both mothers and infants. Policies to promote breastfeeding are well established, and breastfeeding should be encouraged regardless of effects on childhood BMI.^13^ Both breastfeeding and childhood obesity are of increasing interest to bodies such as the National Institute for Health and Care Excellence, the Department of Health, Public Health England, and the National Health Service. Breastfeeding, if found to reduce childhood BMI, could be an important part of wider early life obesity interventions.

There are various theories suggesting the mechanisms by which breastfeeding might influence BMI.^14–16^ In this study, we aim to identify the effects of breastfeeding on childhood BMI rather than to determine the reasons that this relationship might occur.

The ideal way of determining causal treatment effects is by carrying out randomized controlled trials (RCTs). However, RCTs cannot be used to study breastfeeding for ethical and practical reasons. Furthermore, RCT results may not be generalizable to the population because mothers’ behaviour might change as a consequence of participating in a trial estimating the effects of a lifestyle intervention.^17^ Breastfeeding promotion has been investigated in the ‘Promotion of Breastfeeding Intervention Trial’ (PROBIT), a cluster randomized trial.^18^ Adiposity was not one of the original trial outcomes, but subsequent studies investigated the effects of breastfeeding promotion on childhood adiposity.^12,19^ They were limited in that the PROBIT trial only included mothers who intended to breastfeed. They estimated the intention to treat effect (ITE) which only identifies the effect for a subgroup of participants, those who change their behaviour directly as a consequence of the intervention.

For these reasons, observational data have been used as an alternative to randomized data.^20^ However, observational data can suffer from selection bias due to lack of randomization and this must be accounted for appropriately in order to produce reliable estimates. Existing studies have often used regression models, most commonly a linear or logistic regression,^4,5,8–10,21–29^ which make assumptions that have been criticized within the literature.^6,11^ Propensity score matching (PSM) is a technique that tries to mimic a randomized trial while relaxing some of these assumptions. It deals with selection on observable characteristics, does not extrapolate to unobservable parts of the data and avoids imposing a functional form on the relationship between breastfeeding and BMI. Other studies have previously used propensity score (PS) approaches,^11,30,31^ including a generalized PS approach^11^ and inverse probability of treatment weights,^30^ but both of these approaches impose a functional form which is not required when using PSM. Grube *et al*. used PSM to investigate the effects of breastfeeding on childhood overweight and obesity.^31^ They compared children who were breastfed for over 4 months with those breastfed for 4 months or less. The present study estimates a different treatment effect because it uses a different control group (never breastfed) which can be used consistently across a range of breastfeeding treatments. The breastfeeding treatments include different breastfeeding durations, both exclusive and partial. Despite the numerous observational studies in the literature, this study contributes by providing a more extensive analysis which mimics an RCT in an attempt to minimize selection bias. It does not impose a functional form when testing for the differences in mean between the treated and control groups, has a consistent control group and compares a range of breastfeeding treatments.

## Methods

### Data

The Millennium Cohort Study (MCS) contains a rich set of information from a sample of 19 517 children born around the year 2000. Cohort members were recruited using child benefit records (universal at the time) to minimize sample bias. See a report by Plewis^32^ for more details on the MCS, including response rates. The cohort members’ carers were interviewed when the infant was ~9 months old, and detailed information on infant feeding behaviours were recorded. The same children and their carers have since been interviewed at ages 3, 5 and 7 years.^33^ During each of these subsequent interviews, data on height, weight and other physical measures were collected along with detailed information on a variety of socioeconomic and demographic variables allowing a range of potential confounding factors to be accounted for.

#### Outcome variable

Childhood BMI measured at ages 3, 5 and 7 years is calculated using height and weight;

(1) $$\mathrm{BMI}=\frac{\mathrm{weight}\left(\mathrm{kg}\right)}{{\mathrm{height}\left(m\right)}^{2}}$$

Classifications of childhood obesity and overweight are more complex than in adults and there are different definitions. The adiposity rebound^34^ occurs in children around the age of 5 years, after a drop in BMI during early childhood followed by a steady increase in mean BMI until adult definitions can be used.

#### Treatment variables

We explore the effects of a range of breastfeeding ‘treatments’ on childhood BMI at different ages. These breastfeeding treatments differ by exclusivity and duration and are (i) breastfeeding initiation, (ii) partially breastfed for 4 weeks, (iii) partially breastfed for 16 weeks, (iv) exclusively breastfed for 4 weeks and (v) exclusively breastfed for 16 weeks. In each case, infants satisfying the required criteria were considered ‘treated’. They were then compared with children in the control group who were never breastfed. Children who were breastfed but did not meet the treatment criteria were removed from the analysis. This means that the control groups are consistent for each of the binary treatments, keeping analysis as similar as possible to an RCT.

#### Control variables

Control variables that potentially confound the relationship between breastfeeding and childhood BMI were chosen in accordance with existing literature. These variables include high and low maternal education, high and low socioeconomic status, home ownership/tenancy, sex and ethnicity, living with both natural parents, maternal marital status, maternal obesity, mother in care as a child, maternal longstanding illness, whether a pregnancy was planned, maternal age at birth of the child, maternal smoking status during each trimester of pregnancy, alcohol consumption during pregnancy, birth weight, prematurity and the logged length of hospital stay. Variables likely to affect both childhood BMI and the propensity to breastfeed recorded during pregnancy or as close to the time of birth as possible are included in order to predict the propensity to breastfeed. This is in line with the literature which suggests that these variables should be time invariant or measured before the treatment.^35^ It is possible that some of these variables will change over time during childhood and these changes could influence childhood BMI, but not through breastfeeding. In addition, many confounding variables are likely to be highly correlated with each other and so it is not necessary to include all of them in the estimation of the PS because including one will often account for the effect of others. For example, maternal and child diet and exercise will be highly correlated with maternal education, which has already been accounted for. Nevertheless, we will perform robustness checks in order to ensure that any remaining unobserved confounding is minimal.

#### Excluded observations

We exclude the following observations from our analysis. In the second wave, 692 new families (699 children) entered the MCS but breastfeeding information was missing. We exclude children from multiple births due to their different breastfeeding experiences and the potential influences that multiple birth could have on BMI. We also exclude children who weighed <2.5 kg at birth, those who remained in hospital immediately after birth for over 14 days and those with a gestational period <196 days, considered to be ‘extremely preterm’ by WHO^36^. Observations are removed in accordance with the World Health Organization (WHO) recommendations for biologically implausible values; these include childhood and maternal height, weight and BMI. Only observations for which the cohort member's natural mother was interviewed are included due to the lack of information and possible inaccuracy of breastfeeding variables from other carers. Observations with missing values are excluded and assumed to be missing at random. Suitable data were available for a sample of 11 200, 11 744 and 10 707 children at ages 3, 5 and 7, respectively. The number of observations excluded from the sample at each age is available in the appendix.

### Statistical analysis

Using PSM, we compare treatment and control groups, in effect, emulating an RCT. Treated observations are matched to control observations with similar characteristics using a PS. The PS, given observable characters ***X*** is

(2) p(X)=Pr(d=1X)

where d=1 if an observation is treated. The PS, estimated using a probit model, estimates the likelihood of being in the treated group. Matching observations using a PS is equivalent to matching on each observable characteristics.^37^

PSM prevents extrapolation to parts of the relationship, which are not observed in the data, restricting the analysis to the region of ‘common support’, outside of which the treatment and control groups are not balanced potentially causing bias. In addition, PSM imposes no functional form on the relationship between the outcome and treatment. Regression models assume a functional form,^6,11^ which, if incorrect, could lead to biased results.

We use a nearest neighbour algorithm with a calliper to restrict the difference in PS between matched observations. We check for bias by ensuring that each confounder does not significantly differ in mean between the treated and control groups. More discussion of PSM and its assumptions can be found in the literature.^35,37,38^ The strongest assumption of PSM is that there remains no unobserved confounding. It is impossible to prove that no unobserved confounding exists,^39^ but we include a number of sensitivity analyses to assess the robustness of the results.

PSM can provide estimates for the average treatment effect on the treated (ATT), the average treatment effect on the untreated (ATU) and the average treatment effect for the population (ATE). We are interested in the ATE which is most relevant to any population-wide policies^40^ and is most comparable with the existing literature and with RCT estimates. The ATT and ATU are not discussed here but are presented in the appendix.

We used the user-written ‘psmatch2’ command^41^ in Stata 13 and the ‘pstest’ command for post-estimation checks.

## Results

**Fig. 1:**
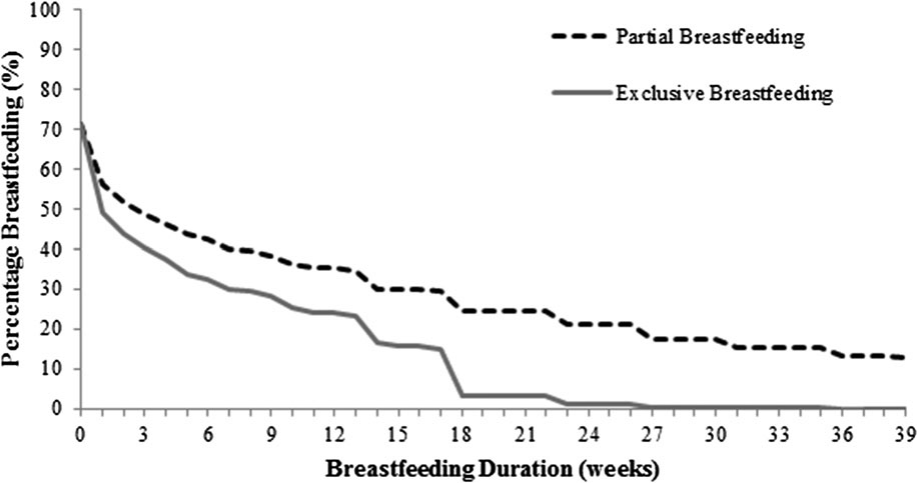
Percentage of cohort members still breastfed by durations.

Table 1 shows the mean BMI and proportions of overweight and obesity^42^ of all children in the samples, as well as for children who were and were never breastfed. The adiposity rebound is apparent by the dip in BMI at 5 years. The prevalence of overweight and obesity consistently increases with age. Figure 1 shows the percentages of cohort members still breastfed, exclusively and partially, by duration. Breastfeeding was initiated in 71% of cohort members. At 4 weeks, <50% of cohort members were partially breastfed and <40% were exclusively breastfed. By 16 weeks (in 2000 the WHO recommended that weaning should start at 16 weeks), these numbers drop to 30% and 16%, respectively.

**Table 1:** Summary statistics for adiposity variables

Variable	3 years	*N*	5 years	*N*	7 years	*N*
BMI (kg/m^2^) mean (standard deviation)	16.78	11 200	16.31	11 744	16.60	10 707
	(1.561)		(1.679)		(2.224)	
Mean BMI in breastfed children	16.74	7794	16.26	8127	16.53	7476
	(1.535)		(1.657)		(2.17)	
Mean BMI in non-breastfed children	16.85	3446	16.44	3617	16.76	3231
	(1.621)		(1.722)		(2.335)	
Overweight^a^ in full sample	23.34%	11 200	21.03%	11 744	20.16%	10 707
Overweight^a^ in breastfed children	22.22%	7794	19.92%	8127	19.05%	7476
Overweight^a^ in non-breastfed children	25.86%	3446	23.53%	3617	22.72%	3231
Obese^a^ in full sample	4.98%	11 200	5.16%	11 744	5.39%	10 707
Obese^a^ in breastfed children	4.76%	7794	4.87%	8127	4.92%	7476
Obese^a^ in non-breastfed children	5.48%	3446	5.81%	3617	6.47%	3231

*Notes:* Mean with standard deviation in parentheses.

^a^Overweight and obesity are defined using the International Obesity Task Force thresholds which vary by age and sex.

The results from probit models used to estimate the propensity of each breastfeeding treatment in the sample of 3 year olds are displayed in Table 2. Results are similar for the samples at other ages suggesting that attrition does not significantly influence the results, similar to other studies’ findings.^32,33^ The sign and significance of the coefficients are as expected and similar to those found in other studies.^20^ Using link tests, we find no evidence of misspecification in these probit models.

**Table 2:** Estimation of PS at age 3 years

	Probit model estimating breastfeeding				
	(1)	(2)	(3)	(4)	(5)
Age	−0.00180	−0.00231	−0.000925	−0.00191	−0.00143
	(0.00124)	(0.00141)	(0.00165)	(0.00148)	(0.00198)
Sex	0.0348	0.0357	0.0249	0.0265	−0.0474
	(0.0268)	(0.0301)	(0.0350)	(0.0314)	(0.0417)
Black	1.246^***^	1.483^***^	1.637^***^	1.244^***^	1.428^***^
	(0.126)	(0.133)	(0.148)	(0.147)	(0.181)
Asian	0.681^***^	0.787^***^	0.852^***^	0.656^***^	0.821^***^
	(0.0572)	(0.0624)	(0.0705)	(0.0667)	(0.0818)
Other	0.756^***^	0.864^***^	0.956^***^	0.788^***^	0.889^***^
	(0.0888)	(0.0976)	(0.111)	(0.103)	(0.131)
High education	0.339^***^	0.365^***^	0.399^***^	0.357^***^	0.426^***^
	(0.0454)	(0.0493)	(0.0553)	(0.0512)	(0.0655)
Low education	−0.254^***^	−0.330^***^	−0.406^***^	−0.334^***^	−0.366^***^
	(0.0384)	(0.0430)	(0.0495)	(0.0447)	(0.0596)
High SES	0.257^***^	0.308^***^	0.340^***^	0.321^***^	0.356^***^
	(0.0458)	(0.0490)	(0.0539)	(0.0508)	(0.0624)
Low SES	−0.274^***^	−0.304^***^	−0.366^***^	−0.293^***^	−0.343^***^
	(0.0325)	(0.0365)	(0.0422)	(0.0380)	(0.0503)
Live with both natural parents	0.276^***^	0.288^***^	0.333^***^	0.263^***^	0.325^***^
	(0.0429)	(0.0505)	(0.0625)	(0.0528)	(0.0779)
Mother married	0.0319	0.0561	0.0470	0.0633	0.110^*^
	(0.0346)	(0.0388)	(0.0451)	(0.0405)	(0.0538)
Home owners	0.0947^*^	0.0948^*^	0.0726	0.0972^*^	0.0527
	(0.0376)	(0.0430)	(0.0509)	(0.0451)	(0.0614)
Private renters	0.180^***^	0.220^***^	0.270^***^	0.223^***^	0.219^*^
	(0.0517)	(0.0595)	(0.0707)	(0.0622)	(0.0871)
Birth weight	−0.0110	−0.00594	0.0301	−0.0108	0.0179
	(0.0276)	(0.0311)	(0.0367)	(0.0328)	(0.0439)
Hospital stay (log)	0.129^***^	0.0948^**^	0.0641	0.0864^**^	0.0442
	(0.0258)	(0.0290)	(0.0340)	(0.0304)	(0.0410)
Planned pregnancy	0.0939^**^	0.108^**^	0.0974^*^	0.0995^**^	0.0583
	(0.0299)	(0.0335)	(0.0388)	(0.0349)	(0.0460)
Premature	−0.0807	−0.0992	−0.245^**^	−0.162^*^	−0.266^**^
	(0.0601)	(0.0684)	(0.0830)	(0.0726)	(0.0995)
Mother obese	−0.0273	−0.110	−0.282^***^	−0.139^*^	−0.379^***^
	(0.0488)	(0.0560)	(0.0685)	(0.0592)	(0.0858)
Mother age at birth	0.0117^***^	0.0247^***^	0.0358^***^	0.0256^***^	0.0433^***^
	(0.00270)	(0.00306)	(0.00359)	(0.00319)	(0.00429)
Smoker first trimester	−0.0790^*^	−0.168^***^	−0.344^***^	−0.183^***^	−0.353^***^
	(0.0335)	(0.0384)	(0.0457)	(0.0400)	(0.0551)
Smoker second trimester	−0.335^***^	−0.415^***^	−0.454^***^	−0.371^***^	−0.577^***^
	(0.0826)	(0.0981)	(0.119)	(0.100)	(0.158)
Smoker third trimester	−0.341^***^	−0.454^***^	−0.652^***^	−0.474^***^	−0.741^***^
	(0.0532)	(0.0633)	(0.0807)	(0.0664)	(0.104)
Alcohol during pregnancy	−0.000174	−0.00106	0.0148	0.000330	0.00984
	(0.0129)	(0.0151)	(0.0169)	(0.0155)	(0.0228)
Mother in care at 16 years	−0.0299	−0.116	−0.146	−0.126	0.123
	(0.132)	(0.162)	(0.210)	(0.171)	(0.233)
Maternal longstanding illness	0.0522	0.0138	−0.0245	−0.0118	−0.120^*^
	(0.0326)	(0.0371)	(0.0435)	(0.0389)	(0.0531)
Caesarean section delivery	−0.118^**^	−0.138^**^	−0.169^***^	−0.178^***^	−0.168^**^
	(0.0382)	(0.0430)	(0.0502)	(0.0455)	(0.0603)
Constant	0.122	−0.372	−1.213^***^	−0.479	−1.632^***^
	(0.247)	(0.281)	(0.331)	(0.294)	(0.395)
*N*	11 200	8845	6949	7885	5290

*Notes:* Standard errors in parentheses. ^*^P < 0.05, ^**^P < 0.01, ^***^P < 0.001. Probit model varying by breastfeeding treatment; these binary treatments are (1) ever breastfed, (2) partially breastfed for 4 weeks, (3) partially breastfed for 16 weeks, (4) exclusively breastfed for 4 weeks and (5) exclusively breastfed for 16 weeks.

We find that at least 80% of eligible observations lie within the common support in each of the matching analyses, more than in similar studies.^20^ Using t-tests, we find that the majority of covariates are balanced between treatment and control groups at a 95% significance level and all are balanced at a 90% significance level. Results are robust to other matching algorithms and other measures of childhood adiposity, including obesity and overweight.

Table 3 shows the ATEs on BMI for different breastfeeding treatments alongside the mean BMI of the unmatched control groups. Breastfeeding initiation appears to reduce childhood BMI in all waves, but its effect is generally small and statistically insignificant until the age of 7 years. Breastfeeding for longer durations reduces BMI to a greater extent for both partial and exclusive breastfeeding, but effects are larger when breastfeeding is prolonged and exclusive. The effects get larger as children get older. By the age of 7 years, children who were exclusively breastfed for 16 weeks benefited from 0.28 kg/m^2^ (95% confidence interval (CI) 0.07 to 0.49) reduction in BMI compared to those who were never breastfed. The mean BMI at 7 years was 16.6 kg/m^2^.

**Table 3:** Average treatment effects using PSM

	Mean BMI in control group	Mean difference in BMI between treated and control (standard error)				
	(0)	(1)	(2)	(3)	(4)	(5)
Age 3	16.86	−0.0392	−0.0333	−0.0086	−0.0602	−0.1592**
		(0.0419)	(0.0470)	(0.0077)	(0.0421)	(0.0785)
*N*	3446	9330	7877	6949	7451	5183
Age 5	16.44	−0.0782	−0.1086**	−0.1772**	−0.1401***	−0.2031**
		(0.0456)	(0.0535)	(0.0686)	(0.0484)	(0.0824)
*N*	3617	9996	6858	4841	7829	5423
Age 7	16.76	−0.1591**	−0.1665**	−0.2416***	−0.2072***	−0.2762**
		(0.0672)	(0.0767)	(0.0761)	(0.0743)	(0.1077)
*N*	3231	8372	6168	6534	7167	4948

*Notes:* Bootstrapped standard errors in parentheses. ^*^P < 0.1, ^**^P < 0.05, ^***^P < 0.01. PSM varying by breastfeeding treatment; these binary treatments are (0) never breastfed: control group, (1) ever breastfed, (2) partially breastfed for 4 weeks, (3) partially breastfed for 16 weeks, (4) exclusively breastfed for 4 weeks and (5) exclusively breastfed for 16 weeks.

We test the underlying assumption of PSM that there remains no unobserved confounding using a two-stage instrumental variable model for each of the breastfeeding treatments at ages 3, 5 and 7 years. We used delivery by caesarean section (or not) as a binary instrument for breastfeeding behaviour^43^ along with Sargan–Hansen *post hoc* tests for any unobserved confounding. We found insufficient evidence to support the existence of remaining confounding. In addition, we jointly estimated BMI and breastfeeding using maximum likelihood in a restricted version of a Roy model.^44,45^ Any correlation between the error terms in these jointly estimated equations would point towards the existence of unobserved confounding, but likelihood ratio tests failed to reject the null hypothesis of no correlation between the error terms using a 95% CI. Based on this evidence, we believe it is reasonable to assume that there are no important confounders that remain unaccounted for and thus PSM is an appropriate technique in this setting where an RCT is not possible.

## Discussion

### Main findings of this study

The results indicate that the effects increase as children get older and when breastfeeding is exclusive or continued for longer durations. Although breastfeeding can produce significant reductions in BMI, the effects appear small. However, these small differences during childhood are likely to lead to larger differences during adulthood. Obese children are more likely to become obese adults.^1^ In addition, the standard deviation of BMI increases with age.^42,46^ This suggests that any differences in mean BMI at young age between the treated and control groups will increase if individuals remain on the same BMI percentile as adults. This is also supported by the increasing effects as children get older, suggesting that the reductions in BMI accumulate throughout early childhood and might take time to be identified. If these reductions in childhood BMI continue to become larger and more significant as children get older, then there could be substantial differences in BMI as a result of breastfeeding by the time a child reaches adolescence or adulthood.

### What is already known on this topic

There is little doubt that breastfeeding and BMI are correlated. The literature is inconclusive about whether this association is causal or whether it can be completely explained by confounding factors. RCTs are not feasible because the well-known benefits of breastfeeding mean that randomization might influence maternal behaviour^17^ causing bias. The closest to an RCT in breastfeeding are the PROBIT trials,^12,18,19^ which randomized breastfeeding promotion. However, this study did not estimate a nationally representative sample and could not identify the ATE of breastfeeding on BMI, only the ITE.

### What this study adds

This study contributes to existing literature by acknowledging the underlying assumptions imposed when estimating the effects of breastfeeding on BMI using observational data. We use PSM in order to prevent extrapolation outside the observed data and to relax the assumptions of functional form imposed by regression models^6^ and other methods involving PS.^11,30^ We also use a more consistent control group than previous studies^31^ in order to compare a range of treatments. We test for unobserved confounding and although it is not possible to prove that unobserved confounding does not exist,^39^ we find no evidence of it. We believe that this study is an improvement on, and produces more conclusive, comprehensive and reliable results, than previous observational studies.

Our results challenge findings from a number of studies that detected no influence of breastfeeding on childhood adiposity^6,8,11,12^ and those that observed an effect which decreased with age.^23^ We find evidence to support studies that found no significant effect on BMI in very young children^5^ and that the correlation between breastfeeding and childhood adiposity is largely attenuated by confounding.^27^

The results support current WHO recommendations for 6 months of exclusive breastfeeding and provide convincing evidence supporting breastfeeding policies, more in line with randomized data. That said, breastfeeding has a limited influence on BMI when used in isolation and should be part of a wider effort to reduce obesity.

### Limitations of this study

The assumption of no unobserved confounding cannot be formally tested;^39^ thus, selection bias might still be present. However, *post hoc* tests find no suggestion of remaining bias.

Children born today might experience different treatment effects to children in this sample, due to, for example, improvements in formula milk and changing attitudes towards breastfeeding. Similarly, increased prevalence of childhood obesity suggests that BMI differences might become visible at a younger age in more recent cohorts. Maternal recall on breastfeeding duration might also effect results but has previously been found to be valid and reliable.^47^

Future research should focus on the effects of breastfeeding on older children and adolescents who are more likely to remain obese throughout adulthood.^48^ Research into how childhood obesity develops over time and its relationship with other lifestyle factors could help us further understand the dynamics of childhood BMI. Additional research is needed into which breastfeeding promotions are most effective and have the greatest long-term impact. Observational studies are likely to play a large part in this research because they provide more long-term data and due to ethical restrictions surrounding breastfeeding.

### Conclusion

We found that the influences of breastfeeding on childhood BMI were significant but unlikely to prevent childhood obesity in isolation. Breastfeeding policies alone cannot solve the obesity epidemic but could be part of wider early-life approaches.

## Supplementary data

Supplementary data are available at the *Journal of Public Health* online.

## Supplementary Material

Supplementary Data
