# Supplementary material for: The effects of breastfeeding on childhood BMI: a propensity score matching approach
Source: J Public Health (Oxf). 2017 Sep 9;39(4):e152–60. doi: 10.1093/pubmed/fdw093 (PMC5939873; doi:10.1093/pubmed/fdw093)
Supplement: Supplementary Data [file fdw093_Supplementary_Data.zip › APPENDIX.docx]

# APPENDIX

**Table 1: Excluded Observations**

| **Variable** | **9 Months** | **3 Years** | **5 Years** | **7 Years** |
| --- | --- | --- | --- | --- |
| Original Sample | 18,552 | 15,808 | 15,460 | 14,043 |
| Late entry | 0 | 699 | 573 | 500 |
| Multiple birth | 256 | 413 | 409 | 351 |
| Mother’s BMI* | 819 | 80 | 666 | 698 |
| Birth weight | 846 | 690 | 747 | 673 |
| Hospital stay | 459 | 362 | 428 | 369 |
| Gestation length | 834 | 679 | 734 | 664 |
| Child’s BMI* | - | 669 | 768 | 683 |
| Number after exclusions (% removed) | 16,219  (12.58) | 13,979  (11.57) | 13,700  (11.38) | 12,494  (11.03) |
| Missing observations | 1,151 | 2,779 | 1,956 | 1,787 |
| # observations in sample  (% of original obs.) | 15,068  (81.22) | 11,200  (70.85) | 11,744  (75.96) | 10,707  (76.24) |

Source: Millennium Cohort Study. Notes: Values are for number of children, not families. Observations can be missing in more than one variable. *implausible or missing height, weight or BMI. Child’s BMI is not measured at nine months.

**Table 2: Propensity Score Matching Results**

| Treatment | BMI of control group^¥^ (s.e.) | # NN  (calliper) | ATT  (s.e.^) | ATT sample size  (com. support) | ATU  (s.e.) | ATU sample size  (com. support) | ATE  (s.e.^€^ ^) | ATE sample size  (com. support) | ATE  95% CI |
| --- | --- | --- | --- | --- | --- | --- | --- | --- | --- |
| **Age 3** | | | | | | | | | |
| Ever breastfed | 16.80 | 1  (0.00024) | -0.0448  (0.0518) | 6,196  (79.9%) | -0.0282  (0.0223) | 3,134  (90.9%) | -0.0392  (0.0419) | 9,330  (83.3%) | (-0.1214,  0.0430) |
| **Partial Breastfeeding** | | | | | | | | | |
| > 4 weeks compared to never breastfed | 16.78 | 2  (0.0005) | -0.0174  (0.0585) | 4,724  (87.5%) | -0.0570*  (0.0298) | 3,153  (91.5%) | -0.0333  (0.0470) | 7,877  (89.1%) | (-0.1254,  0.0589) |
| > 16 weeks compared to never breastfed | 16.80 | 3  (0.0005) | -0.0088  (0.0087) | 2,835  (80.9%) | -0.0083  (0.0068) | 2,700  (78.4%) | -0.0086  (0.0077) | 5,602  (80.2%) | (-0.2291,  -0.0013) |
| **Exclusive breastfeeding** | | | | | | | | | |
| > 4 weeks compared to never breastfed | 16.76 | 3  (0.001) | -0.0512  (0.0580) | 4,178  (94.1%) | -0.0231  (0.0318) | 3,279  (95.2%) | -0.0388  (0.0465) | 7,457  (94.6%) | (-0.1446,  0.0204) |
| > 16 weeks compared to never breastfed | 16.70 | 3  (0.01) | -0.1310*  (0.0790) | 1,822  (98.8%) | -0.1746**  (0.0768) | 3,361  (97.5%) | -0.1592**  (0.0785) | 5,183  (98.0%) | (-0.3131,  -0.0054) |
| **Age 5** | | | | | | | | | |
| Ever breastfed | 16.32 | 1  (0.00025) | -0.0837  (0.0535) | 6,726  (82.8%) | -0.0669**  (0.0294) | 3,270  (90.4%) | -0.0782  (0.0456) | 9,996  (85.1%) | (-0.1675,  0.0112) |
| **Partial** **Breastfeeding** | | | | | | | | | |
| > 4 weeks compared to never breastfed | 16.32 | 2  (0.00025) | -0.0977*  (0.0569) | 4,080  (72.0%) | -0.1246*  (0.0749) | 2,778  (76.8%) | -0.1086**  (0.0535) | 6,858  (73.9%) | (-0.2135,  0.0036) |
| > 16 weeks compared to never breastfed | 16.27 | 3  (0.0003) | -0.1809***  (0.0651) | 2,439  (66.6%) | -0.1735**  (0.0722) | 2,402  (66.4%) | -0.1772**  (0.0686) | 4,841  (66.5%) | (-0.3117,  -0.0428) |
| **Exclusive breastfeeding** | | | | | | | | | |
| > 4 weeks compared to never breastfed | 16.31 | 3  (0.0009) | -0.1623***  (0.0597) | 4,363  (94.0%) | -0.1121***  (0.0342) | 3,466  (95.8%) | -0.1401***  (0.0484) | 7,829  (94.8%) | (-0.2349,  -0.0453) |
| > 16 weeks compared to never breastfed | 16.27 | 3  (0.01) | -0.2176***  (0.0794) | 1,883  (97.9%) | -0.1954**  (0.0840) | 3,540  (97.9%) | -0.2031**  (0.0824) | 5,423  (97.9%) | (-0.3646,  -0.0415) |
| **Age 7** | | | | | | | | | |
| Ever breastfed | 16.67 | 1  (0.0002) | -0.1880**  (0.0773) | 5,565  (74.4%) | -0.1019**  (0.0472) | 2,807  (86.9%) | -0.1591**  (0.0672) | 8,372  (78.2%) | (-0.2908,  0.0274) |
| **Partial Breastfeeding** | | | | | | | | | |
| > 4 weeks compared to never breastfed | 16.65 | 2  (0.00025) | -0.1542*  (0.0841) | 3,697  (70.1%) | -0.1850***  (0.0656) | 2,471  (76.5%) | -0.1665**  (0.0767) | 6,168  (8,474%) | (-0.3168,  -0.0162) |
| > 16 weeks compared to never breastfed | 16.56 | 3  (0.0003) | -0.2139**  (0.1019) | 3,360  (98.5%) | -0.2709***  (0.0488) | 3,174  (98.2%) | -0.2416***  (0.0761) | 6,534  (98.4%) | (-0.3908,  -0.0924) |
| **Exclusive breastfeeding** | | | | | | | | | |
| > 4 weeks compared to never breastfed | 16.65 | 3  (0.001) | -0.1845**  (0.0867) | 4,062  (94.2%) | -0.2370***  (0.0581) | 3,105  (96.1%) | -0.2072***  (0.0743) | 7,167  (95.0%) | (-0.3528,  -0.0616) |
| > 16 weeks compared to never breastfed | 16.59 | 3  (0.01) | -0.3674***  (0.1131) | 1,762  (98.2%) | -0.2258**  (0.1047) | 3,186  (98.6%) | -0.2762**  (0.1077) | 4,948  (98.4%) | (-0.4873,  -0.0652) |

Source: Millennium Cohort Study. ^¥^Average BMI in the control group in the sample estimating ATE. ^€^bootstrap standard error (500 repetitions). ^Standard errors assume propensity score is known. Notes: * *p* < 0.05, ** *p* < 0.01, *** *p* < 0.001. Callipers chosen using trial and error to minimise bias and variance. ATT – average treatment effect on the treated, ATU – average treatment effect on the untreated, ATE – average treatment effect (overall).

**Table 3: Estimation of Propensity Scores at Age 3 Years**

|  | Probit model estimating Breastfeeding | | | | |
| --- | --- | --- | --- | --- | --- |
|  | (1) | (2) | (3) | (4) | (5) |
| Age | -0.00180 (0.00124) | -0.00231 (0.00141) | -0.000925 (0.00165) | -0.00191 (0.00148) | -0.00143 (0.00198) |
| Sex | 0.0348 (0.0268) | 0.0357 (0.0301) | 0.0249 (0.0350) | 0.0265 (0.0314) | -0.0474 (0.0417) |
| Black | 1.246^***^ (0.126) | 1.483^***^ (0.133) | 1.637^***^ (0.148) | 1.244^***^ (0.147) | 1.428^***^ (0.181) |
| Asian | 0.681^***^ (0.0572) | 0.787^***^ (0.0624) | 0.852^***^ (0.0705) | 0.656^***^ (0.0667) | 0.821^***^ (0.0818) |
| Other | 0.756^***^ (0.0888) | 0.864^***^ (0.0976) | 0.956^***^ (0.111) | 0.788^***^ (0.103) | 0.889^***^ (0.131) |
| high education | 0.339^***^ (0.0454) | 0.365^***^ (0.0493) | 0.399^***^ (0.0553) | 0.357^***^ (0.0512) | 0.426^***^ (0.0655) |
| low education | -0.254^***^ (0.0384) | -0.330^***^ (0.0430) | -0.406^***^ (0.0495) | -0.334^***^ (0.0447) | -0.366^***^ (0.0596) |
| high SES | 0.257^***^ (0.0458) | 0.308^***^ (0.0490) | 0.340^***^ (0.0539) | 0.321^***^ (0.0508) | 0.356^***^ (0.0624) |
| low SES | -0.274^***^ (0.0325) | -0.304^***^ (0.0365) | -0.366^***^ (0.0422) | -0.293^***^ (0.0380) | -0.343^***^ (0.0503) |
| live with both natural parents | 0.276^***^ (0.0429) | 0.288^***^ (0.0505) | 0.333^***^ (0.0625) | 0.263^***^ (0.0528) | 0.325^***^ (0.0779) |
| mother married | 0.0319 (0.0346) | 0.0561 (0.0388) | 0.0470 (0.0451) | 0.0633 (0.0405) | 0.110^*^ (0.0538) |
| home owners | 0.0947^*^ (0.0376) | 0.0948^*^ (0.0430) | 0.0726 (0.0509) | 0.0972^*^ (0.0451) | 0.0527 (0.0614) |
| private renters | 0.180^***^ (0.0517) | 0.220^***^ (0.0595) | 0.270^***^ (0.0707) | 0.223^***^ (0.0622) | 0.219^*^ (0.0871) |
| birth weight | -0.0110 (0.0276) | -0.00594 (0.0311) | 0.0301 (0.0367) | -0.0108 (0.0328) | 0.0179 (0.0439) |
| hospital stay (log) | 0.129^***^ (0.0258) | 0.0948^**^ (0.0290) | 0.0641 (0.0340) | 0.0864^**^ (0.0304) | 0.0442 (0.0410) |
| planned pregnancy | 0.0939^**^ (0.0299) | 0.108^**^ (0.0335) | 0.0974^*^ (0.0388) | 0.0995^**^ (0.0349) | 0.0583 (0.0460) |
| Premature | -0.0807 (0.0601) | -0.0992 (0.0684) | -0.245^**^ (0.0830) | -0.162^*^ (0.0726) | -0.266^**^ (0.0995) |
| mother obese | -0.0273 (0.0488) | -0.110 (0.0560) | -0.282^***^ (0.0685) | -0.139^*^ (0.0592) | -0.379^***^ (0.0858) |
| mother age at birth | 0.0117^***^ (0.00270) | 0.0247^***^ (0.00306) | 0.0358^***^ (0.00359) | 0.0256^***^ (0.00319) | 0.0433^***^ (0.00429) |
| smoker 1^st^ trimester | -0.0790^*^ (0.0335) | -0.168^***^ (0.0384) | -0.344^***^ (0.0457) | -0.183^***^ (0.0400) | -0.353^***^ (0.0551) |
| smoker 2^nd^ trimester | -0.335^***^ (0.0826) | -0.415^***^ (0.0981) | -0.454^***^ (0.119) | -0.371^***^ (0.100) | -0.577^***^ (0.158) |
| smoker 3^rd^ trimester | -0.341^***^ (0.0532) | -0.454^***^ (0.0633) | -0.652^***^ (0.0807) | -0.474^***^ (0.0664) | -0.741^***^ (0.104) |
| alcohol during pregnancy | -0.000174 (0.0129) | -0.00106 (0.0151) | 0.0148 (0.0169) | 0.000330 (0.0155) | 0.00984 (0.0228) |
| mother in care at 16 years | -0.0299 (0.132) | -0.116 (0.162) | -0.146 (0.210) | -0.126 (0.171) | 0.123 (0.233) |
| maternal longstanding illness | 0.0522 (0.0326) | 0.0138 (0.0371) | -0.0245 (0.0435) | -0.0118 (0.0389) | -0.120^*^ (0.0531) |
| Caesarean Section delivery | -0.118^**^ (0.0382) | -0.138^**^ (0.0430) | -0.169^***^ (0.0502) | -0.178^***^ (0.0455) | -0.168^**^ (0.0603) |
| Constant | 0.122 (0.247) | -0.372 (0.281) | -1.213^***^ (0.331) | -0.479 (0.294) | -1.632^***^ (0.395) |
| *N* | 11200 | 8845 | 6949 | 7885 | 5290 |

Source: Millennium Cohort Study. Notes: Standard errors in parentheses. ^*^ *p* < 0.05, ^**^ *p* < 0.01, ^***^ *p* < 0.001. Probit model varying by breastfeeding treatment; these binary treatments are (1) ever breastfed, (2) partially breastfed for four weeks, (3) partially breastfed for sixteen weeks, (4) exclusively breastfed for four weeks, (5) exclusively breastfed for sixteen weeks.
